# The Small Round Blue Cell Tumors of the Sinonasal Area: Histological and Immunohistochemical Findings

**DOI:** 10.5812/ircmj.4735

**Published:** 2013-06-05

**Authors:** Mohammad J Ashraf, Leila Beigomi, Negar Azarpira, Bita Geramizadeh, Bijan Khademi, Afsoon Hakimzadeh, Elham Abedi

**Affiliations:** 1 Department of Pathology, Shiraz Medical School, Shiraz University of Medical Sciences, Shiraz, IR Iran; 2 Department of Otolaryngology, Shiraz Medical School, Shiraz University of Medical Sciences, Shiraz, IR Iran

**Keywords:** Carcinoid Tumor, IHC64, Histological Techniques

## Abstract

**Background:**

Primary Small round blue cell tumors (SRBCT) in sinonasal comprise histogenetically diverse entities with overlapping morphologic features. Because of the limited initial biopsy tissue materials, differential diagnostic difficulties may arise, and as they have different management, exact diagnosis and classification are very important.

**Objectives:**

In this study, we analyzed the immunohistochemical expression of a panel of markers in the classification and diagnosis of sinonasal SRBCTs.

**Material and Methods:**

This cross sectional study was performed on 36 paraffin embedded tissue samples. Histologic and immunohistochemical slides from 36 patients with SRBCT were analyzed retrospectively. The patients were admitted in Khalili hospital, Shiraz from 1383 to 1388.

**Results:**

There were 13 women and 23 men with the mean age of 53 ±12.1. There were 9 malignant melanoma, seven poorly differentiated SCC; six lymphoma (DLBL); 4 SCNEC; three SNUC; two ON; two Ewing/PNET; two embryonal rhabdomyosarcoma, and one plasmacytoma. Pan-cytokeratin was strongly expressed poorly differentiated SCC and all cases of SNUC. Coexpression of desmin and nuclear myoD1 was only detected in rhabdomyosarcoma. HMB45 was only expressed in sinonasal melanoma. CD99 expression was identified only in Ewing/PNET. FLI-1 was detected in 50% of PNET. P63 was expressed in poorly differentiated SCC (2/7) and SNUC (1/3).

**Conclusions:**

The results of our study indicate that the integration of histopathologic findings with application of limited but highly specific markers led to the separation of carcinomas, lymphoma and melanomas from other small cell tumors. Using a panel of keratin, LCA, desmin, and HMB45 is the most practical and economic approach to accurately classify these tumors.

## 1. Background

The sinonasal regions are host to a variety of malignant neoplasms. Approximately 0.2–0.8 percent of all malignant tumors occur in nasal cavity and paranasal sinuses, and because of the close anatomic relation with the orbits and skull base, disease extension into these structures usually occurs (1, 2). The most common locations are maxillary sinus, followed by the nasal cavity, the ethmoid, sphenoid, and frontal sinuses (1, 2). The ‘‘small round blue cell tumors’’ (SRBCTs) constitute a heterogeneous group of malignant neoplasms characterized by a monomorphic population of undifferentiated cells with small-sized nuclei and scant cytoplasm (1). An early and accurate diagnosis is imperative for appropriate treatment. However, definitive diagnosis of SRBCT based solely on the H & E light microscopic findings may be difficult. Furthermore, pathologist usually receives a small or limited size biopsy, which complicates the diagnosis. Ancillary studies such as immunohistochemistry (IHC), cytogenetic and molecular techniques may be used to roll out differential diagnoses of SRBCTs. The SRBCTs of sinonasal area are categorized as: 

1. Epithelial SRBCTs including Poorly Differentiated, Nonkeratinizing Squamous Cell Carcinoma, Sinonasal Undifferentiated Carcinoma (SNUC), Small Cell Carcinoma, and Neuroendocrine Type (SCCNET) (1, 3, 4). Squamous cell carcinoma (SCC) is the most common malignancy of the sinonasal tract. Well-differentiated and/or keratinizing form of SCC is easily recognizable but the poorly differentiated, non-keratinizing variant may exhibit histopathologic features that overlap with other SRBCTs. Finding of an in situ carcinoma and/or direct continuity of neoplastic cells to the overlying surface epithelium are useful histological finding in favor of epithelial origin. Immunohistochemically, pan cytokeratin, CK7, CK8, and EMA immunoreactivity are useful for distinguishing this neoplasm from other small cell tumors (1, 3, 4). SNUC is a rare, highly aggressive carcinoma which typically presents with locally extensive disease (1, 2). Histopathologically, the tumor cells grow along the mucosal surface epithelium with extension into superficial mucosal glands (1, 2). Individual malignant cells exhibit hyperchromatic to vesicular nuclei with high nuclear-to-cytoplasmic ratio and prominent nucleoli. Immunohistochemically, the tumor cells are immunoreactive for pan-cytokeratins and simple keratins with no amplification of Epstein-Barr virus (EBV) RNA by in situ hybridization (1, 3, 4). SCCNETs are composed of small sized cells with oval or round hyperchromatic nuclei and absent or inconspicuous nucleoli, arranged in sheets, nests, and/or trabeculae. Crush artifact with a high mitotic rate is a common finding (1, 2, 5). Punctuate perinuclear cytokeratin staining is an important finding. CD56 staining is also common (1, 5).

2. Neuroectodermal SRBCTs including Olfactory Neuroblastoma (ON), Sinonasal Mucosal Malignant Melanoma, and Extraskeletal Ewing’s sarcoma/Primitive Neuroectodermal Tumor (ES/PNET). Olfactory neuroblastoma (ON) is uncommon, accounting for only 1% to 5% of malignant nasal cavity neoplasms (6-8). ON is thought to originate from the olfactory portion of the mucous membrane lining the nasal fossa and is virtually confined to the upper nasal cavity in the region of the cribriform plate. The presence of fibrillary cell processes, Homer Wright rosettes, and S100-positive sustentacular cells are histological findings in favor of ON. In contrast to PNET, ON is not immunoreactive for CD99 (6, 8). Amelanotic variant with small cell morphology of malignant melanomas is prone to misclassification with other SRBCTs of this area. Melanin pigment is observed in approximately 2/3 of cases (7, 9). Diffuse staining for S-100 and HMB45 are useful for final diagnosis (1, 7, 10). CD99 immunoreactivity is useful in distinguishing ES/PNET from most other SRBCTs. Identification of the characteristic t (11;22)(q24;q12) or EWSR1-FLI1 fusion transcript or defined variant translocation can be invaluable in confirming this diagnostic entity (11).

3. Mesenchymal SRBCTs including Rhabdomyosarcoma, Poorly Differentiated Synovial Sarcoma. Both embryonal and alveolar rhabdomyosarcoma frequently occur in the sinonasal area (1, 2). Rhabdomyosarcoma usually express myogenic markers such as MyoD1 and desmin. However aberrant staining for CK and synaptophysin is reported. Distinguishing from SCCNET or SNUC is possible with identification of the 2;13 and 1;13 translocations or respective PAX3-FOXO1 and PAX7-FOXO1 fusion transcripts (1, 2). Poorly Differentiated Synovial Sarcoma rarely occurs in sinonasal tract. Immunoreactivity for cytokeratin, EMA, BCL2, coupled with cytogenetic or FISH confirmation of the t (X;18) (p11.2;q11.2) or derived SYT-SSX chimeric transcripts facilitates the diagnosis (1, 2, 12).

4. Hematolymphoid SRBCTs including Extramedullary Plasmacytoma, Extranodal NK/T Cell Lymphoma (Nasal-Type) and Diffuse large B cell lymphoma (DLBL) (1, 13). In respect of extramedullary plasmacytoma, when tumor is mainly composed of mature plasma cell the diagnosis is not challenging. But in poorly differentiated tumors (immature plasma cells) with occasional EMA or cytokeratin positivity, misdiagnosed as a carcinoma may occur. The second most common malignancy of the sinonasal tract following SCC is malignant lymphoma (13, 14). Extranodal NK/T Cell Lymphoma (Nasal-Type) frequently affects Asian and Latin American populations (13). Demonstration of the EBV virus (EBV-encoded early RNAs) by in situ hybridization in addition to a NK-cell immunophenotype (CD3-, CD56+, perforin and granzyme B+) confirmed the diagnosis (13-15). DLBL is typically more common in Western populations. Immunophenotypically, DLBL has positive findings for B-lineage markers (CD20+, CD79+, CD3-, CD56) (1, 12).

## 2. Objectives 

In this study, we analyzed the immunohistochemical expression of a panel of markers in the classification and diagnosis of sinonasal SRBCTs.

## 3. Patients and Methods

We retrieved 36 cases of SRBCT from division of surgical pathology archives at the Shiraz University of Medical Sciences, between 2006 and 2011. All hematoxylin and eosin–stained and immunohistochemical slides of these tumors were available and reevaluated independently by two head and neck pathologists. The final diagnoses were rhabdomyosarcoma, olfactory neuroblastoma, Ewing sarcoma, sinonasal undifferentiated carcinomas (SNUCs), neuroendocrine carcinomas, and sinonasal melanomas. No original diagnoses were changed on rereview. Table 1 presents the markers used and the conditions for the IHC staining procedure. Immunohistochemical markers included known lineage-specific markers (pan cytokeratin AE1/AE3, S100, HMB45, desmin, and myoD1), basal cell marker P63, markers of neuroendocrine differentiation (chromogranin, synaptophysin, and NSE), markers for lymphoma (leukocyte common antigen (LCA), CD20, CD3). The FLI-1, product of chromosomal translocation between the FL1 gene and EWS gene, are considered for ancillary marker of Ewing/PNET Markers were evaluated for cytoplasmic staining except for P63, S100, LIF-1 and myoD1, which had positive results for nuclear expression, and CD99, for which membranous staining was considered.

**Table 1.** Immunohistochemical markers and their conditions used in the tissue diagnosis

Manufacture	Chromogene	Retrieval	Dilution	Clone	Biomarker
**Dako, Denmark**	DAB, Envision	Citrate	Ready to use	AE1/AE3	*CK*
**Dako, Denmark**	DAB, Envision	Citrate	Ready to use	-	*S100*
**Novacastra**	DAB, Envision	Citrate	1/50	-	*HMB45*
**Dako, Denmark**	DAB, Envision	Citrate	Ready to use	V9	*Vimentin*
**Dako, Denmark**	DAB, Envision	Citrate	1/100	2B11	*LCA*
**Dako, Denmark**	DAB, Envision	Citrate	1/75	D33	*Desmin*
**Dako, Denmark**	DAB, Envision	Citrate	1/200	5.8A	*MyoD1*
**Dako, Denmark**	DAB, Envision	Citrate	Ready to use	BBs/NC	*NSE*
**Dako, Denmark**	DAB, Envision	TE	Ready to use	-	*Chromogranin*
**Dako, Denmark**	DAB, Envision	Citrate	Ready to use	SY38	*Synaptophysin*
**Dako, Denmark**	DAB, Envision	Citrate	1/200	12E7	*CD99*
**Novacastra**	DAB, Envision	Citrate	1/100	-	*P63*
**Santa Cruz, Santa Cruz, CA, USA**	DAB, Envision	Citrate	1/400	sc-356	*LFI-1(C-19)*

## 4. Results

### 4.1. Age and Sex Distribution

There were 13 women and 23 men, age range from 2 to 84 years, with a median age of 53 years. The peak incidence was during the sixth and seventh decades of life in both sexes. Men represented a higher proportion of tumors than women, with male/female ratio of 2/1.

### 4.2. Histologic Distribution

**Figure 1.**
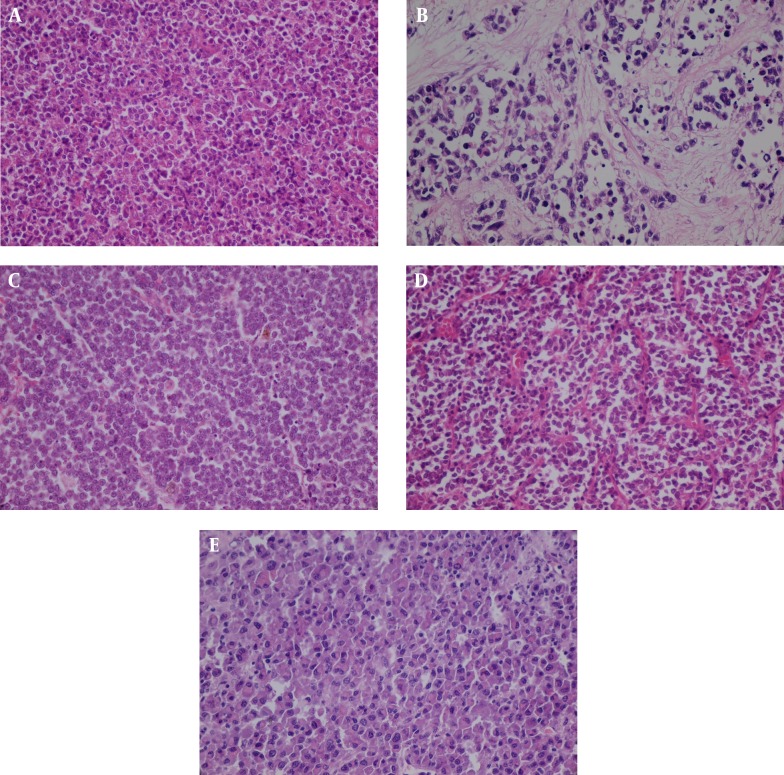
Histological Comparison of Small Round Blue Cell Tumors in Sinonasal Area A: Lymphoma, B: Rhabdomyosarcoma, C: Melanoma, D: PNET, E: Plasmacytoma

There were nine malignant melanoma, seven poorly differentiated SCC, six lymphoma (DLBL), four SCNEC, three SNUC, two ON, two Ewing/PNET, two embryonal rhabdomyosarcoma, and one plasmacytoma (Table 1) (Figure 1A-E).

### 4.3. Immunohistochemical Analysis

**Figure 2.**
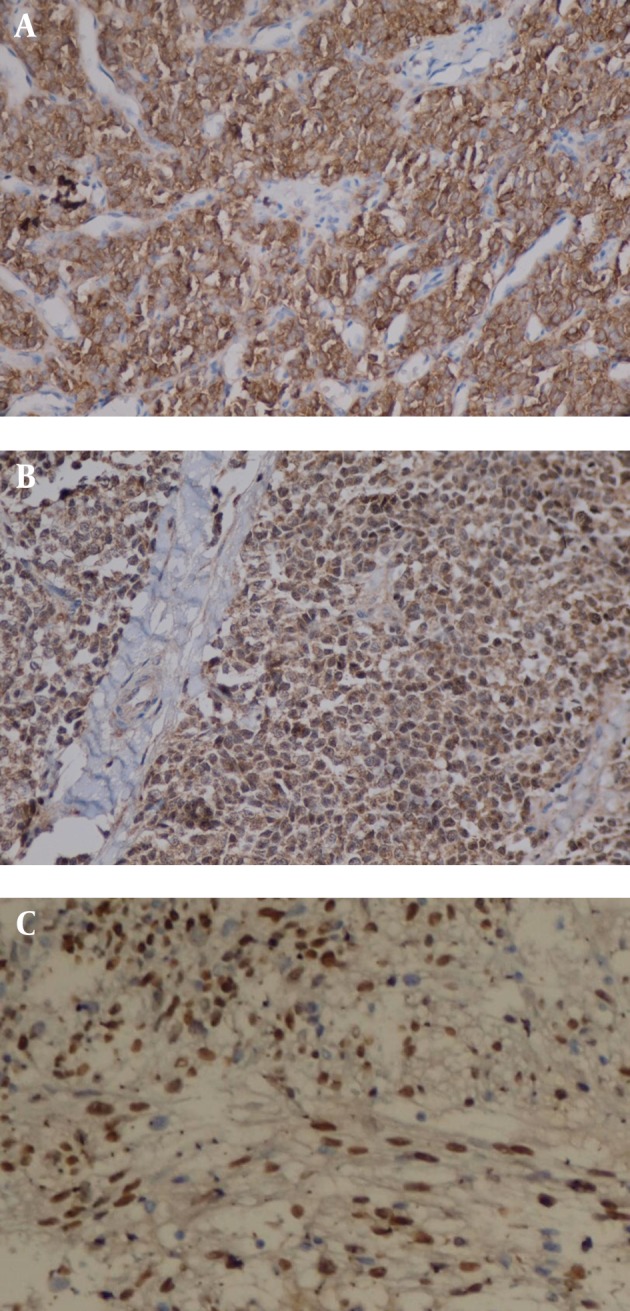
Immunohistochemical Staining of Small Round Blue Cell Tumors in Sinonasal Area A: Membranous Staining of CD99 in PNET, B: Nuclear FLi-1 Staining in PNET, C: Desmin Expression in Rhabdomyosarcoma

The IHC staining results for sinonasal tumors are presented in Table 2 and patterns of expression were shown in Figure 2 A-C

**Table 2.** Expression of immunohistochemical markers in sinonasal tumors

FLI-1	P63	CD99	Synapto^a^	Chromo^a^	NSE	MyoD1	Desmin	LCA	Vimentin	CK^a^	HMB45	S100	Number	Tumor type
** 0/9 **	0/9	0/9	0/9	0/9	0/9	0/9	0/9	0/9	0/9	0/9	9/9	8/9	9(27)	Sinonasal melanoma
** 0/7 **	2/7	0/7	0/7	0/7	0/7	0/7	0/7	0/7	0/7	7/7	0/7	0/7	7(19.4)	Poorly differentiated SCC
** 1/6 **	0/6	0/6	0/6	0/6	0/6	0/6	0/6	6/6	0/6	0/6	0/6	0/6	6 (16.7)	Lymphoma
** 0/4 **	0/4	0/4	4/4	4/4	3/4	0/4	0/4	0/4	2/3	2/4	0/4	0/4	4(11.1)	SCNEC^a^
** 0/3 **	1/3	0/3	0/3	0/3	0/3	0/3	0/3	0/3	0/3	3/3	0/3	0/3	3(8.3)	SNUC^a^
** 0/2 **	0/2	0/2	1/2	1/2	2/2	0/2	0/2	0/2	0/2	0/2	0/2	0/2	2(5.6)	ON^a^
** 1/2 **	0/2	2/2	0/2	0/2	0/2	0/2	0/2	0/2	2/2	0/2	0/2	1/2	2(5.6)	Ewing/PNET
** 0/2 **	0/2	0/2	0/2	0/2	0/2	2/2	2/2		2/2	0/2	0/2	0/2	2(5.6)	Embryonal rhabdo
** 0/1 **	0/1	0/1	0/1	0/1	0/1	0/1	0/1	1/1	0/1	0/1	0/1	0/1	1(2.8)	Plasmacytoma

^a^abbreviations: Chromo, chromogranin; CK, cytokeratin; Synapto, synaptophysin; SNUC, sinonasal undifferentiated carcinoma. SCNEC, Small cell neuroendocrine carcinoma; ON, Olfactory neuroblastoma Final diagnoses based on immunohistochemical and morphologic assessment.

### 4.4. A: Epithelial Markers

Pan-cytokeratin was strongly expressed in 12 (33%) of the 36 tumors; included in all cases poorly differentiated SCC (7/7), all SNUCs (3/3), and 2 of 4 SCNEC.

### 4.5. B: Neuroendocrine Markers

Neuroendocrine markers (chromogranin, synaptophysin and NSE) were collectively expressed in 12 (33.3%) of the 36 tumors. Synaptophysin and chromogranin had positive findings in all 2 SCNEC and 1 of 2 ON. NSE expression was noted in 2 of 2 ON, and 3 of 4 SCNEC. Coexpression of two or three of these markers was found in most neuroblastomas and SCNEC. 

### 4.6. C: Skeletal Muscle Markers

Of the 36 tumors, 2 (5.5%) had positive results for desmin and coexpressed nuclear myoD1, confirmed the diagnosis of rhabdomyosarcoma.

### 4.7. D: Melanocytic Markers

S100 was expressed in 10 (28%) of 36 tumors. Expression was identified in 8 of 9 sinonasal melanomas, and showed focally dispersed positive cells in PNET (1/2). HMB45 was only expressed in sinonasal melanomas (9/9).

### 4.8. E: Lymphoma Markers

LCA and CD20 were expressed in 6 (16.7%) of 36 tumors. All of them were diagnosed as non-Hodgkin lymphoma, BLBL with further IHC markers.

### 4.9. F: Miscellaneous New Markers

CD99 is a sialomucin glycoprotein adhesion molecule, and is used as a marker in the diagnosis of Ewing/PNET tumors. CD99 expression was identified only in Ewing/PNET tumors (2/2). FLI-1, a marker for Ewing sarcoma, was present in 2 (5.5%) of 36 tumors. It was expressed in Ewing/PNET (1/2), and 1 of 6 lymphoma. The P63, a marker for primitive basal cells, was present in 3 (8.3%) of 36 tumors. It was expressed in poorly differentiated SCC (2/7) and 1 of 3 SNUC.

## 5. Discussion

SRBCTs of the sinonasal tract are rare tumors with a wide spectrum of biological activity and diverse clinical behavior. In the present study, these neoplasms usually occurred in the sixth and seventh decades of life, and rarely found in young age group. This finding was consistent with the results of the literature review in which the average median age of patients in the reported series was 57 years (Table 2) ( 12 , 15 , 16  ). In our series, the male/ female ratio was 2/1. In the literature review, it was mentioned that men represented a higher proportion of malignant neoplasms than women, with male/female ratio of 1.8 (range from 1.2 to 5.3) ( 17 - 20 ). The combined light microscopic rereview immunophenotyping and molecular findings led to the confirmation of the diagnosis in all of the 36 tumors. Aberrant expression of certain histogenetic marker was not detected in this study. Epithelial tumors (carcinoma) constitute most of sinonasal neoplasms. In the literature, carcinomas composed of differentiated SCC, nonkeratinizing poorly differentiated SCC, SNUC and SCCNET ( 1 , 2 , 16 , 21 ). However, in our study, carcinomas constituted 39% of all sinonasal SRBCTs and differentiated SCC was not considered at all. Overexpression of P63 was considered as a marker for squamous differentiation ( 21 ). However, better differentiated areas and even well-differentiated tumors may be negative ( 10 , 21 , 22 ). In our study, P63 was founded in two of seven cases of poorly differentiated SCC. In the study performed on sinonasal SRBCTs by Wooff et al. ( 23 ), 63 had positive results in all cases of nonkeratinizing , poorly differentiated SCC (2 of 2), and in single cases of mantle cell lymphoma (1 of 1) and poorly differentiated neuroendocrine carcinoma (1 of 1); however, it inconsistently stained diffuse large B-cell lymphoma (4 of 5), extranodal NK/T-cell lymphoma, nasal type (1 of 4), sinonasal undifferentiated carcinoma (1 of 6), and Ewing sarcoma/primitive neuroectodermal tumor (2 of 6). These differences may be explained by variability at two phases of the procedure: (1) the antibody may not detect all P63 isoforms (2), the difference in interpretation. The first possibility is less likely. Because most of the IHC studies use antibodies that detect all P63 isoforms (TAp63α, TAp63β, TAp63γ, ΔNp63α, ΔNp63β, ΔNp63γ) ( 10 , 21 , 23 , 24 ). In agreement with other authors, we considered a positive result when equal or more than 50% of tumor cells was intensely stained with antibody ( 21 ). This discrepancy could be attributed to the use of a small core of tissue and the variable processing differences of the assembled blocks. In the Ewing case, the initial diagnosis was most likely based on the detection of CD99 in tumor cells ( 25 ). But, nonspecific diagnostic nature of CD99 was reported in several tumor studies ( 25 - 27 ). In our study, no aberrant CD99 expression was found and diffuse strong membranous staining was detected only in Ewing/PNET. Combination of FLI-1 polyclonal (FLI-1p) and CD99 was considered as a valuable immunohistochemical approach for the diagnosis of EWS ⁄ PNET. Mhawech-Fauceglia et al., believed that the best antibody combination for the diagnosis of EWS ⁄ PNET was CD99 and FLI-1p (C-19, polyclonal, Santa Cruz, USA), with a very high sensitivity ( 26 , 27 ). However, these two markers can be expressed in other neoplasms, including vascular tumors and Merkel cell carcinoma of the skin ( 28 ). We had only two cases of Ewing/PNET, both of them had positive results for CD99, but the LFI-1 was stained in one case. Due to low case number, the correlation between CD99 and FLI-1p was not possible. The application of molecular analysis of the EWS-FLI1 fusion transcript was suggestive of Ewing sarcomas and PNET confirmation. Melanoma was the major malignancy in our case, more than reported frequency by Cordes et al. ( 16 ). Like previous studies, our data showed a high incidence of S100 expression in sinonasal melanomas ( 29 ). The S100 was not expressed in one case; therefore, we advocate concurrent use of other melanoma markers (HMB-45, Melan- A). Similarly, S100 may also play a role in the diagnosis of ON, but no expression was found in our two cases. Therefore it must be used in conjunction with other complementary markers ( 30 ). Lymphomas, manifested as infiltrative sheet-like growth of CD45 positive tumor cells and easily separated for other sinonasal small cell tumors ( 2 , 14 ). The results of our study indicate that the integration of histopathologic findings with a panel of selected lineage specific markers is necessary for early diagnosis and classification of SRBCTs of sinonasal area. The initial application of limited but highly specific markers led to the separation of carcinomas, lymphoma and melanomas from other small cell tumors. Using a panel of keratin, LCA, desmin, and HMB45 is the most practical and economic approach to accurately classify these tumors ( 10 , 31 - 33 ). A second order of complimentary markers supported the initial diagnosis in most tumors and confirmed the histogenesis of few other cases. Molecular markers such as EWS-FLI1 and PAX-FKHR may be valuable for diagnostic confirmation of Ewing/PNET and rhabdomyosarcoma. Another entity named as NUT midline carcinoma should be considered if undifferentiated carcinomas with focal squamous differentiation arise in the sinonasal area ( 34 , 35 ). This neoplasm, uniquely characterized by rearrangements of the NUT or 15q14 gene (q14;p13.1). The nuclear expression of NUT can be detected by immunohistochemistry, fluorescence in situ hybridization (FISH) or reverse transcriptase–polymerase chain reaction (RT-PCR) ( 34 , 35 ). SRBCTs arising in the sinonasal are heterogeneous and accurate classification may be challenging due to overlapping histopathologic features. This study provides an integrative and organized approach for the application of a limited set of markers for diagnosis of these tumors. 
